# PrEP uptake preferences among men who have sex with men in China: results from a National Internet Survey

**DOI:** 10.1002/jia2.25242

**Published:** 2019-02-06

**Authors:** Jing Han, Jennifer ZH Bouey, Liming Wang, Guodong Mi, Zihuang Chen, Ying He, Tara Viviani, Fujie Zhang

**Affiliations:** ^1^ Beijing Ditan Hospital Capital Medical University Beijing China; ^2^ Clinical Center for HIV/AIDS Capital Medical University Beijing China; ^3^ Department of International Health SNHS Georgetown University Washington DC USA; ^4^ Blued Beijing China; ^5^ Department of Biology Georgetown University Washington DC USA

**Keywords:** PrEP uptake, PrEP knowledge, young Chinese MSM, HIV risk behaviours, HIV prevention

## Abstract

**Introduction:**

HIV incidence among men who have sex with men (MSM) is high in China. Pre‐exposure prophylaxis (PrEP) is a promising mean to prevent HIV transmission but it is not widely available in China. We conducted a large Internet‐based online survey to assess the willingness of Chinese MSM to take PrEP and associated factors to their uptake preferences.

**Methods:**

Between 19 January and 6 February, 2017, 4581 MSM aged over 15 years were recruited via a social networking app to take an online PrEP survey. HIV status at the time of the survey being conducted was not one of recruitment criteria. Participants were asked if they had heard of PrEP, if they had concerns about PrEP, and if they would be ready to uptake PrEP should it be provided. When asked if participants were willing to take PrEP, they were asked to select from the following responses: “definitely not,” “probably not,” “not sure,” “probably yes,” and “definitely yes.” In the final analysis, we grouped these five‐level Likert scale responses into three‐level responses as “definitely yes,” “probably yes,” and “no (definitely not/probably not/not sure).” Descriptive analysis and multinomial logistic regressions were conducted to assess the associations of PrEP adoption readiness and uptake concerns with HIV risk behaviours and demographic characteristics.

**Results:**

MSM from 33 geographical regions of China participated in the survey. The majority were younger than 25 (65.2%) and had attended college (68.6%). HIV prevalence was high (6.8%) and 43.3% reported a history of unprotected anal sex. Only 22.4% of participants had heard of PrEP. When asked if they would uptake PrEP, 26.0% said “definitely yes,” 49.6% were “probably yes,” and 24.4% said “no.” PrEP adoption readiness was associated with having previously heard of PrEP and expressing concerns about accessibility and cost. Worries about side effects, low perceived HIV risk, preference for condoms, and never having received HIV testing were negatively associated with PrEP uptake willingness.

**Conclusion:**

Young and well‐educated Chinese MSM reported a low willingness to uptake PrEP despite being high‐risk for HIV. Effective education, especially through online mediums, will be critical to optimize this group's PrEP uptake.

## 1 Introduction

The National HIV Sentinel Surveillance in China revealed that the prevalence of human immunodeficiency virus (HIV) among Chinese men who have sex with men (MSM) has increased steadily from 1.5% in 2005 to 8.0% in 2015 1. The urban MSM appear to be susceptible to even higher prevalence estimates as a 2017 multi‐city study reported a 9.9% prevalence rate 2. In some cities in particular, HIV prevalence is as high as 14% 3 to 19.7% 4 among MSM. Additionally, MSM comprise over a quarter of China's newly diagnosed cases 5, which is more than three times the rate of new diagnoses for MSM in other countries 6. In light of these trends, there is an urgent need for China to implement HIV prevention measures among MSM.

Pre‐exposure prophylaxis (PrEP), a daily, oral antiretroviral medication, has emerged as one of the most promising means of preventing HIV transmission 7 and has recommended by the United States Centers for Disease Control (US CDC) and the World Health Organization (WHO) for HIV prevention 8, 9, 10, 11. While the medication is clearly effective, the fact remains that its success is ultimately dependent on its adoption 12, 13. PrEP uptake has been shown to be both feasible and promising in studies among MSM in North America 14, Africa 15, and India 16, but further analyses are needed in other countries, including China.

Our study used a National Internet Survey to examine factors associated with the acceptability of PrEP among Chinese MSM. We chose this study design because China is the world's largest Internet and mobile phone market and numbered close to 700 million users in 2017 17, 18. Internet‐based surveys are also attractive to young MSM who regularly use their mobile phones for dating and face a disproportionately high risk for HIV infection 19, 20, 21. Guided by a MSM community organization and primarily administered through a National MSM social networking site, our survey drew a large sample size, from which we assessed demographic characteristics, high‐risk sexual behaviours and PrEP knowledge.

## 2 Methods

### 2.1 Recruitment and data collection

Between 19 January 2017 and 6 February 2017, an Internet‐based survey was administrated on PrEP knowledge and acceptability among Chinese MSM. The survey featured 30 questions and took approximately 10 minutes to complete (please see Appendix S1 for the survey questionnaire). Participants were recruited primarily through banner advertisements on “BLUED,” a common MSM dating App in mainland China with over 27 million registered Chinese users 22.

For eligibility, participants had to reside in China, be at least 15 years of age, and being MSM. We aimed to assess the overall PrEP uptake preferences among the whole MSM community, therefore, we did not exclude HIV positives from our study population. Those interested were directed to a webpage survey questionnaire. All were informed that participation was voluntary and anonymous by clicking a button to read the online consent form. Study protocol was approved by the Institutional Review Board at Beijing Ditan Hospital, Beijing, China (no. IRB00004487).

### 2.2 Measures

Participants were presented with a scenario in which PrEP was readily available and asked a number of survey‐specific questions about whether they would take PrEP for HIV prevention, how they would prefer to access PrEP, and if they had any concerns regarding PrEP. To assess the willingness of PrEP, participants were asked to select from the following responses: “definitely not,” “probably not,” “not sure,” “probably yes” and “definitely yes.” In the final analysis, we grouped these five‐level Likert scale responses into three‐level responses as “definitely yes,” “probably yes” and “no (definitely not/probably not/not sure).” Participants were also asked to choose the type of venue they would like to visit to obtain PrEP if they chose willingness to receive PrEP as “definitely yes” and “probably yes.” Participants were asked to select reasons they would be reluctant to take PrEP if they selected “no” to receive PrEP.

Participants were asked if they had heard of PrEP before. If they answered “yes,” they were asked to select where they obtained that information from. Additionally, they were asked to indicate who they thought should take PrEP. Questions about PrEP knowledge were developed specifically for this survey.

To assess associations of HIV risk behaviours, participants were asked how many male sexual partners they had in the last 12 months and whether they had engaged in unprotected anal sexual intercourse (UAI). If the participant had engaged in UAI, he was asked how many partners he had participated in UAI within the last 12 months and if he had ever engaged in UAI with a stranger. Participants were also asked if they had been tested for HIV and other sexual transmitted infections (STIs), when they had last been tested, what their HIV/STI statuses were, and whether they normally ask sexual partners about HIV. All survey questions regarding HIV risk behaviours and testing were drawn from those employed in the Chinese National HIV sentinel surveillance system, which is the main source for Chinese HIV epidemic estimates and reports 23, 24.

### 2.3 Demographics

Measured demographic characteristics included sexual orientation, age, place of birth, place of residence, marital status, education level, annual income, and gender identity (at birth and at present).

Demographic survey questions were borrowed from the Chinese National HIV sentinel surveillance system 23, 25.

### 2.4 Data analysis

Only those who met our inclusion criteria were included in the final analysis. For the unadjusted analysis, Chi‐square (χ^2^) tests were used to examine differences in PrEP uptake preferences and PrEP awareness across demographic characteristics, HIV risk and testing behaviours, and PrEP concerns. We used multinomial logistic regression models to calculate the adjusted odds ratios, based on a three‐level PrEP uptake preference of “no” (reference group), for MSM who reported “probably yes” and MSM who reported “definitely yes” to PrEP. The adjusted odds ratios describe the multiplicative effect of a unit increase in each predictor on the odds of selecting “definitely yes” or “probably yes” for PrEP uptake compared to the base response group that selected “no.” After checking for collinearity risks, we built a model with predictors of PrEP awareness, HIV risk behaviours, HIV testing status, and general PrEP concerns that was adjusted for age, income, and sexual orientation. All statistical analyses were conducted using STATA version 14.0 (StataCorp LP, College Station, TX, USA).

## 3 Results

### 3.1 Demographic characteristics and HIV risks

In total, 4581 Chinese MSM enrolled in the final analysis. These participants represented all of the China's 33 geographical divisions: 22 provinces, four municipalities, five autonomous regions, and Macau and Taiwan (please see Appendix S2 for details). The most represented regions included Guangdong province (n = 456), Shandong province (n = 314), Beijing (n = 300), Henan province (n = 286) and Jiangsu (n = 248). Table 1 presents the demographic characteristics and HIV risks of the Chinese MSM who completed the survey as well as the association of those characteristics with PrEP awareness and uptake preferences.

**Table 1:** Demographic and HIV risks among men who have sex with men (MSMs) who participated in the China MSMs HIV PrEP Uptake Willingness Online Survey, 2017 (N = 4581)

	Total	Heard of PrEP			PrEP uptake preference			
		Yes (%)	No (%)	χ[Link]	Definitely yes (%)	Probably yes (%)	No (%)	χ[Link]
	(N = 4581)	(n = 1028)	(n = 3553)	*p* value	(n = 1191)	(n = 2274)	(n = 1116)	*p* value
Age group				<0.001				<0.001
<18	486 (10.6)	76 (7.4)	410 (11.5)		120 (10.1)	240 (10.6)	126 (11.3)	
18 to 25	2501 (54.6)	538 (52.3	1963 (55.2)		583 (49.0)	1276 (56.1)	642 (57.5)	
26 to 30	908 (19.8)	249 (24.2)	659 (18.5)		252 (21.2)	443 (19.5)	213 (19.1)	
31 to 40	533 (11.6)	141 (13.7)	392 (11.0)		172 (14.4)	251 (11.0)	110 (9.9)	
>40	153 (3.3)	24 (2.3)	129 (3.6)		64 (5.4)	64 (2.8)	25 (2.2)	
Marital status				0.173				0.004
Single	3603 (78.6)	802 (78.0)	2801 (78.8)		902 (75.7)	1798 (79.1)	903 (80.9)	
Married with a woman	410 (9.0)	80 (7.8)	330 (9.3)		130 (10.9)	203 (8.9)	77 (6.9)	
Cohabitation with a man	416 (9.1)	110 (10.7)	306 (8.6)		107 (9.0)	211 (9.3)	98 (8.8)	
Cohabitation with a woman	25 (0.6)	7 (0.7)	18 (0.5)		8 (0.7)	7 (0.3)	10 (0.9)	
Separated, divorced or widowed	127 (2.8)	29 (2.8)	98 (2.8)		44 (3.7)	55 (2.4)	28 (2.5)	
Educational attainment				<0.001				0.001
Junior high or lower	306 (6.7)	43 (4.2)	263 (7.4)		84 (7.1)	120 (5.3)	102 (9.1)	
High school	1134 (24.8)	229 (22.3)	905 (25.5)		303 (25.4)	555 (24.4)	276 (24.7)	
College and above	3141 (68.6)	756 (73.5)	2385 (67.1)		804 (67.5)	1599 (70.3)	738 (66.1)	
Annual income (RMB)				<0.001				0.004
<10,000	1768 (38.6)	342 (33.3)	1426 (40.1)		409 (34.3)	924 (40.6)	435 (39.0)	
10,001 to 30,000	895 (19.5)	191 (18.6)	704 (19.8)		240 (20.2)	419 (18.4)	236 (21.1)	
30,001 to 150,000	1681 (36.7)	428 (41.6)	1253 (35.3)		465 (39.0)	823 (36.2)	393 (35.2)	
150,001 and above	237 (5.2)	67 (6.5)	170 (4.8)		77 (6.5)	108 (4.7)	52 (4.7)	
Sexual orientation				<0.001				<0.001
Homosexual	3170 (69.2)	757 (73.6)	2413 (67.9)		869 (73.0)	1576 (69.3)	725 (65.0)	
Bisexual	1065 (23.3)	217 (21.1)	848 (23.9)		257 (21.6)	530 (23.3)	278 (24.9)	
Not sure	346 (7.6)	54 (5.3)	292 (8.2)		65 (5.5)	168 (7.4)	113 (10.1)	
Had unprotected anal sex last 12 months				0.246				<0.001
Yes	1984 (43.3)	429 (41.7)	1555 (43.8)		604 (50.7)	949 (41.7)	431 (38.6)	
No	2597 (56.7)	599 (58.3)	1998 (56.2)		587 (49.3)	1325 (58.3)	685 (61.4)	
Had HIV testing				<0.001				<0.001
<1 year	2284 (49.9)	648 (63.0)	1636 (46.0)		705 (59.2)	1072 (47.1)	507 (45.4)	
1 to 5 years	378 (8.3)	96 (9.3)	282 (7.9)		100 (8.4)	204 (9.0)	74 (6.6)	
Never	1919 (41.9)	284 (27.6)	1635 (46.0)		386 (32.4)	998 (43.9)	535 (47.9)	

χ^2^, Chi‐squared test; HIV, human immunodeficiency virus; PrEP, pre‐exposure prophylaxis.

The majority of participants were 18 to 25 years old (54.6%), single (78.6%) and had attended some form of college or university (68.6%). About a quarter of respondents (25.5%) identified as migrant, and an overwhelming majority were of the Han ethnicity (92.3%). When asked about their sexual orientation, 69.2% identified themselves as homosexual, 23.3% identified as bisexual and 7.6% were not sure. An annual income of <10,000 RMB was earned by 38.6% of survey participants but a similar proportion, 36.7%, reported earning between 30,001 and 150,000 RMB per year.

While nearly half of the sample (49.9%) had HIV testing in the last year, 41.9% reported never having been tested for HIV. Among those who did receive testing, 156 (6.8%) said they were HIV positive. A large proportion of participants, 43.3%, reported engaging in unprotected anal sex in the past 12 months (Table 1).

### 3.2 PrEP awareness

Only 1028 of the 4581 respondents (22.4%) had heard of PrEP before taking the survey. Compared to their peers, those between the ages of 26 and 40 who were of a higher education levels, belonged to a greater income bracket, or identified as homosexual rather than bisexual or nonconforming were significantly more likely to report having heard of PrEP (Table 1). Those who had HIV testing were also more likely to report having heard of PrEP compared to those who had never been tested (25.4% to 28.4% vs. 14.8%, *p* < 0.001). HIV risk behaviour, ethnicity, migrant status, and marital status were not associated with self‐reported PrEP awareness (Table 1).

Among those who had heard of PrEP, 668 (65.0%) learned about the medication from the Internet, whereas 24.3% received information from contacts in the MSM community. Only 10.7% of those who knew of PrEP received information in a medical facility. Compared to those who had not heard of PrEP, MSM with prior knowledge of the drug were more likely to say “definitely yes” to PrEP uptake (32.6% vs. 24.1%, *p* < 0.001). They were also more likely to prefer to obtain the medication from medical facilities (21.5% vs. 13.9%, *p* < 0.001) than from pharmacies or community organizations (Table 2). Those who had heard of PrEP were also less likely to doubt PrEP's efficacy (50% vs. 58%, *p* < 0.001), or worry about having access to PrEP (38.2% vs. 43.9%, *p* < 0.001). They were, however, more likely to be concerned about the financial burden of adopting and adhering to PrEP (47.6% vs. 43.9%, *p* < 0.001) (Table 2).

**Table 2:** PrEP uptake preferences and PrEP awareness predicted by the PrEP concerns among men participated in the China MSMs HIV PrEP Uptake Willingness Online Survey, 2017 (N = 4581)

	Total (N = 4581)	Heard of PrEP			PrEP uptake preferences			
		Yes (%) (n = 1028)	No (%) (n = 3553)	χ² *p* value	Definitely yes (%) (n = 1191)	Probably yes (%) (n = 2274)	No (%) (n = 1116)	χ²*p* value
PrEP uptake preferences				<0.001				
Yes	1191 (26.0)	335 (32.6)	856 (24.1)					
Maybe	2274 (49.6)	486 (47.3)	1788 (50.3)					
No	1116 (24.4)	207 (20.1)	909 (25.6)					
Heard of PrEP								<0.001
Yes	1028 (22.4)				335 (28.1)	486 (21.4)	207 (18.5)	
No	3553 (77.6)				856 (71.9)	1788 (78.6)	909 (81.5)	
Preferences on where to get PrEP								0.294
Medical facilities	713 (15.5)	221 (21.5)	492 (13.8)	<0.001	249 (20.9)	464 (20.4)		
Pharmacies/Internet pharmacies/vending machine	2121 (46.3)	452 (44.0)	1669 (47.0)		710 (59.6)	1411 (62.0)		
Gay communities	581 (12.7)	138 (13.4)	443 (12.5)		210 (17.6)	371 (16.3)		
Others	1166 (25.5)	217 (21.1)	949 (26.7)		22 (1.8)	28 (1.2)		
Concerns about PrEP								
Perceived risk of HIV				0.102				<0.001
Low	903 (19.7)	221 (21.5)	682 (19.2)		143 (12.0)	408 (17.9)	352 (31.5)	
High	3678 (80.3)	807 (78.5)	2871 (80.8)		1048 (88.0)	1866 (82.1)	764 (68.5)	
Doubts on PrEP's efficacy				<0.001				0.061
Yes	2604 (56.8)	514 (50.0)	2090 (58.8)		646 (54.2)	1328 (58.4)	630 (56.5)	
No	1977 (43.2)	514 (50.0)	1463 (41.2)		545 (45.8)	946 (41.6)	486 (43.5)	
Worry about PrEP's side effects				0.476				<0.001
Yes	3203 (69.9)	728 (70.8)	2475 (69.7)		803 (67.4)	1661 (73.0)	739 (66.2)	
No	1378 (30.1)	300 (29.2)	1078 (30.3)		388 (32.6)	613 (27.0)	377 (33.8)	
Financial burden				0.036				<0.001
Yes	2048 (44.7)	489 (47.6)	1559 (43.9)		602 (50.5)	1005 (49.1)	441 (39.5)	
No	2533 (55.3)	539 (52.4)	1994 (56.1)		589 (49.5)	1269 (50.1)	675 (60.5)	
Have no access to PrEP				0.001				<0.001
Yes	1954 (42.7)	393 (38.2)	1561 (43.9)		588 (49.4)	973 (42.8)	393 (35.2)	
No	2627 (57.3)	635 (61.8)	1992 (56.1)		603 (50.6)	1301 (57.2)	723 (64.8)	
Inconvenience in taking PrEP everyday				0.554				0.565
Yes	1078 (23.5)	249 (24.2)	829 (23.3)		281 (23.6)	547 (24.1)	250 (22.4)	
No	3503 (76.5)	779 (75.8)	2724 (76.7)		910 (76.4)	1727 (75.9)	866 (77.6)	
Prefer using condom as protection for HIV				0.050				<0.001
Yes	1734 (37.9)	416 (40.5)	1318 (37.1)		321 (27.0)	889 (39.1)	524 (47.0)	
No	2847 (62.1)	612 (59.5)	2235 (62.9)		870 (73.0)	1385 (60.9)	592 (53.0)	

MSM, men who have sex with men; χ^2^, Chi‐squared test; HIV, human immunodeficiency virus; PrEP, pre‐exposure prophylaxis.

### 3.3 Uptake preferences and concerns

Of 4581 MSM included in this study, 26.0% said they would use PrEP 49.6% indicated that they were “probably yes” ready to adopt PrEP and 24.4% said they were not ready to adopt PrEP (Table 2). When asked where they would most like to access PrEP, the majority of MSM responded that they preferred pharmacies, Internet pharmacies or vending machines (46.3%), whereas 15.6% opted for medical facilities, 12.7% selected for gay communities and 25.45% indicated they would prefer somewhere other than the aforesaid locations. Over half of participants had concerns about PrEP, including doubts of PrEP's efficacy (56.8%) and worries about PrEP's side effects (69.9%). Other concerns included the financial burden associated with PrEP (44.7%) as well as if they would have access to PrEP (42.7%) (Table 2).

Earlier we described that participants who had heard of PrEP were more likely to select “definitely yes” when asked if they were willing to uptake PrEP (Table 2). In contrast, those who perceived a low risk of HIV were less likely to say “definitely yes” to adopting PrEP (15.8% vs. 28.5%, *p* < 0.001), along with those who preferred to use condoms for HIV prevention (18.5% vs. 30.6%, *p* < 0.0010), and those who were worried about PrEP's side effects (25.1% vs. 28.5%, *p* < 0.001). On the other hand, concerns about financial burden or lack of access to PrEP were associated with increased willingness of participants to adopt PrEP (29.4% vs. 23.3% and 30.1% vs. 23.0%, *p* < 0.001) (Table 2).

### 3.4 Uptake‐adjusted multivariable model

Table 3 presents adjusted odds ratios (AORs) of variables potentially associated with PrEP uptake preferences among MSM. Even after adjusting for HIV risk behaviour, HIV testing status and demographic characteristics, participants who had heard of PrEP were more likely to say “definitely yes” (AOR = 1.7, 95% CI: 1.4 to 2.2) and “probably yes” to PrEP uptake (AOR = 1.2, 95% CI: 1.0 to 1.5) compared to those who had never heard of PrEP. Similarly, those who reported UAI in the last 12 months had a tendency to say “definitely yes” (AOR = 1.4, 95% CI: 1.1 to 1.5) and “probably yes” (AOR = 1.1, 95% CI: 1.0 to 1.5) to PrEP uptake compared to those who did not report UAI.

**Table 3:** Adjusted ORs for PrEP knowledge and PrEP uptake preferences among men participated in the China MSMs HIV PrEP Uptake Willingness Online Survey, 2017 (N = 4581)

	PrEP uptake	
	“Definitely yes” AOR (95% CI)	“Probably yes” AOR (95% CI)
Heard of PrEP		
No	1.0	1.0
Yes	**1.7 (1.4 to 2.2)**	**1.2 (1.0 to 1.5)**
Had unprotected anal sex last 12 months		
No	1.0	1.0
Yes	**1.4 (1.1 to 1.6)**	**1.1 (1.0 to 1.5)**
Had HIV testing		
<1 year	1.0	1.0
1 to 5 years	0.9 (0.7 to 1.3)	**1.3 (1.0 to 1.7)**
Never	**0.7 (0.5 to 0.8)**	1.0 (0.8 to 1.2)
Concerns about PrEP (reference)		
Perceived risk of HIV (perceived high risk)	**0.3 (0.3 to 0.4)**	**0.5 (0.4 to 0.6)**
Doubts on PrEP's efficacy (no doubt)	0.8 (0.7 to 1.0)	0.9 (0.8 to 1.1)
Worry about PrEP's side effects (no worries)	0.9 (0.7 to 1.1)	**1.3 (1.1 to 1.5)**
Financial burden (no financial burden)	**1.4 (1.1 to 1.6)**	1.1 (0.9 to 1.3)
Have no access to PrEP (had access to PrEP)	**1.7 (1.4 to 2.0)**	**1.4 (1.2 to 1.6)**
Inconvenience in taking PrEP everyday (convenience)	1.0 (0.8 to 1.3)	1.1 (0.9 to 1.3)
Prefer using condom as protection for HIV (do not want to use condom)	**0.5 (0.4 to 0.5)**	**0.7 (0.6 to 0.9)**
Age group		
<18	1.0	1.0
18 to 25	0.9 (0.6 to 1.1)	1.1 (0.8 to 1.4)
26 to 30	1.0 (0.7 to 1.4)	1.1 (0.8 to 1.5)
31 to 40	1.2 (0.8 to 1.7)	1.1 (0.8 to 1.6)
>40	**2.0 (1.1 to 3.6)**	1.3 (0.7 to 2.2)
Annual income (RMB)		
<10,000	1.0	1.0
10,001 to 30,000	0.9 (0.7 to 1.2)	0.8 (0.6 to 1.0)
30,001 to 150,000	1.0 (0.8 to 1.3)	0.9 (0.7 to 1.1)
150,001 and above	1.3 (0.8 to 1.9)	0.9 (0.6 to 1.3)
Sexual orientation		
Homosexual	1.0	1.0
Bisexual	0.9 (0.7 to 1.1)	0.9 (0.8 to 1.1)
Not sure	0.6 (0.4 to 0.9)	0.7 (0.6 to 1.0)

AOR, adjusted odds ratio; CI, confidence interval; HIV, human immunodeficiency virus; MSM, men who have sex with men; OR, odds ratio; PrEP, pre‐exposure prophylaxis.

Bold values indicated the figure has statistical significance.

After adjusting for PrEP awareness, UAI and demographic characteristics, HIV testing behaviour was associated with willingness to take PrEP in our survey population. Compared to those who had HIV testing in the past year, participants who had never tested for HIV were less likely to say “definitely yes” to PrEP uptake (AOR = 0.7, 95% CI: 0.5 to 0.8). Participants who had HIV testing in last 5 years were more likely to response “probably yes” when asked about taking PrEP (AOR = 1.3, 95% CI: 1.0 to 1.7).

Participants who had a low self‐perceived risk of HIV were less likely to say “definitely yes” (AOR = 0.3, 95% CI: 0.2 to 0.4) or “probably yes” (AOR = 0.5, 95% CI: 0.4 to 0.6) to PrEP uptake. A similar trend was found among those who preferred condoms as their primary form of HIV prevention (AOR = 0.5, 95% CI: 0.4 to 0.5) (Table 3). Worries about PrEP side effects prompted participants to be more likely to say “probably yes” to PrEP uptake compared to those without these concerns (AOR = 1.3, 95% CI: 1.1 to 1.5), but worries about side effects did not significantly impact likelihood to say “definitely yes” to PrEP uptake (AOR = 0.9, 95% CI: 0.7 to 1.1). Finally, we found that those who had concerns about financial burdens and access to PrEP were more likely to say “definitely yes” to the PrEP uptake compared to those who did not voice those concerns (AOR = 1.4, 95% CI: 1.1 to 1.6). In the adjusted model, being over 40 years old was associated with PrEP uptake (AOR = 2.0, 95% CI: 1.1 to 3.6), whereas participants who were “not sure” about their sexual orientation were less likely to be willing to take PrEP (AOR = 0.6, 95% CI: 0.4 to 0.9) (Table 3).

## 4 Discussion

Our study is the first to assess the PrEP uptake preferences and associated factors with a National Internet Survey. The 4581 MSM who participated represent every province in mainland China. The majority of participants were young adults aged 18 to 25 years old. Education levels of these young men were also much higher than the National average 26. This unprecedented geographical representation of young, highly educated MSM suggests a general acceptance of Internet‐based health and behavioural surveys in China.

Our survey identified a number of HIV risk behaviours common among survey participants, including high self‐reported rates of UAI (43.3%) and low levels of HIV and other STI testing. These results shed light on the findings of other studies in China that have reported alarmingly high prevalence rate of HIV, including 6.8%, 7.0% and 7.75% among MSM 1, 27, 28. These results highlight the need for better prevention strategies as well as the urgency of understanding the potential challenges that may complicate prevention initiatives in this high‐risk community 2, 29.

The majority of our survey participants doubted PrEP and only a quarter (26%) selected that they would be willing to take PrEP despite the fact that it is one of the most promising modes of HIV prevention. Most studies in China have focused on rates of PrEP acceptance rather than actual uptake 30, 31, 32, 33, 34, and while reported levels of PrEP acceptance among Chinese MSM vary widely from 19.1% to 71.3% 30, 32, 33, 34, 35, uptake has been consistently reported as very low 31, 36. Compared to younger MSM, our study identified those who were over 40 years old had higher rate of PrEP uptake. This finding is similar to that of Ding *et al*. who found that MSM over 45 were more likely to take PrEP than those between the ages of 18 and 24 (AOR = 2.18, 95% CI: 1.13 to 4.23) 35. One possible explanation is that younger MSM are more sensitive to concerns about PrEP's side effects than older MSM given they face a longer duration of treatment 35. We also found high financial burden and high concern about the access to PrEP among the group of MSM willing to uptake PrEP when available. This may indicate that those who are concerned about accessibility hope the Chinese government will provide PrEP for free, similar to the free antiretroviral therapy (ART) currently provided by the government.

Our study further provides important insights into the role education may play in advancing PrEP acceptability and the success of combined HIV prevention approaches. First, we observed that participating MSM who had previously heard of PrEP were much more likely to say “definitely yes” to adopting the medication and much less likely to express efficacy concerns. However, only 22.4% of our survey participants had heard of PrEP before taking the survey. Low levels of PrEP awareness have been consistently reported in the Chinese MSM community 30, 31. Although many studies have demonstrated that oral Tenofovir‐based PrEP regimens are effective and safe among MSM 37, a considerable proportion of our sample was concerned about PrEP's efficacy (56.8%) and potential side effects (69.9%). These two concerns emerged as more notable barriers to PrEP adoption within our study sample than within other study samples in Asia. In particular, studies conducted among female and transgender sex workers have reported that only 28.4% and 36.8% of those unwilling to use PrEP were weary of side effects 34, 38. A likely contributor to the lack of sufficient HIV and PrEP knowledge is the stigmatization of MSM in China, which prevents the community from accessing HIV resources and care services 39. An effective roll‐out of PrEP in the Chinese MSM community will therefore require broader educational campaigns to increase PrEP knowledge as well as efforts to reduce HIV stigma 39.

Our study sample was also highly educated and regularly accessed information on mobile phones, which suggests that there is great potential for educational and promotional online PrEP campaigns to successfully reach Chinese MSM 17, 18. In fact, MSM who participated in our survey actually indicated that they would trust the Internet more than medical facilities or community organizations for information on PrEP. In addition, a high proportion of our survey participants had heard of PrEP online and preferred “Internet pharmacies, normal pharmacies or vending machines” as their chosen mode of PrEP acquisition. Our survey also clearly indicated that MSM in China prefer the Internet to medical facilities when looking for general health information. Therefore, we conclude that online educational efforts can greatly facilitate PrEP uptake among young Chinese MSM 17. Those who saw themselves as high risk for HIV (39.0% vs. 20.8%) and those who did not think condom use offered sufficient HIV protection (30.2% vs. 20.8%) were also more likely to say they were willing to adopt PrEP. This is similar to findings of Draper *et al*. 40, who observed a 10 to 20% increase in PrEP willingness among those who perceived themselves as having a high HIV risk compared to those who did not. Consequently, in targeted HIV prevention and PrEP promotion campaigns, it may be valuable to use statistical evidence to emphasize the high rates of HIV in MSM populations and increase their perceived levels of HIV risks.

Our study is drawn from a large and targeted data set covering most geographical areas in China and includes the results of the first online survey of its kind among Chinese MSM. However, our study was not exempt from limitations, the most important of which was the use of a cross‐sectional survey and its natural reliance on self‐reported information. Using China's largest MSM dating site as our survey platform, our sample was likely biased towards young MSM, potentially limiting the generalizability of our findings. However, given HIV incidence and the prevalence rates in China are highest among MSM under the age of 25 years of age 41, our survey allowed us to obtain a representative sample of Chinese MSM most vulnerable for HIV. Second, the fact that our survey was administered online limits our study sample to one of convenience and without a specific sampling frame. As a result, our findings may not be reliably applied to MSM who reside in underrepresented provinces. Additionally, this was a cross‐sectional study, no causal relationships can be made. We also did not focus on certain subgroups, such as HIV positive/negative MSM or those who were more likely to exhibit HIV risk behaviours because we aimed to assess overall PrEP uptake preferences among the whole MSM community online. In‐depth analyses into high‐risk subgroups will be conducted later. Finally, our survey did not collect information related to PrEP adherence, which is essential to preventing HIV. This limitation can also be found in other PrEP studies in China as PrEP is not yet widely available. Further studies are needed to assess factors influencing PrEP adherence once the medication is widely available in the country as one study found that only one‐third of trial participants had adherence ≥80% 42. Despite these limitations, our findings illustrate that while young Chinese MSM are at higher risk for HIV, their higher education levels and propensity to use Internet for information and socialization offer a unique opportunity for the implementation of PrEP and other HIV prevention scale‐ups.

## 5 Conclusions

Our findings suggest that MSM in China face a heightened risk of HIV and are in urgent need of effective, preventative health interventions. The PrEP adoption facilitators and barriers we identified in this population prompt us to advocate for a rapid expansion of PrEP advertising and informational campaigns tailored to Chinese MSM. The aims of such programmes should include boosting knowledge of PrEP's effectiveness and side effects as well as increasing awareness of the government's plans facilitate access to PrEP. Public health professionals should also try to address the medical mistrust that exists within this high‐risk population while promoting the use of the Internet as an accurate and confidential source of PrEP information. Finally, HIV prevention cannot be successful without the support and collaboration of the MSM community. Future HIV prevention campaigns should build on the country's successful ART roll‐out, optimize PrEP information through practical and familiar MSM channels, and establish innovative, MSM friendly public health platforms.

## Competing Interest

All authors do not have any commercial or other associations that might pose a conflict of interest.

## Authors’ contributions

JH, LW, and FZ designed the study. JH, LW, GM, ZC, and FZ developed the study protocol including development the data collection tool and study analytic plan. JH, LW, GM, ZC, and FZ were responsible for critical review of the study protocol, carrying out of the study, conducting the data collection, data quality control and data management. JB and YH performed the data cleaning and statistical analysis. TV, JH, and JB wrote the first draft of the manuscript. All other authors revised the first draft and provided critical review to subsequent drafts. All authors have read and approved the final manuscript to be submitted.

## Supporting information


**Appendix S1**: Pre‐exposure prophylaxis(PrEP) knowledge and attitude survey among MSM.

**Appendix S2**: Numbers of participants by provinces/regions.
