# Supplementary material for: PrEP uptake preferences among men who have sex with men in China: results from a National Internet Survey
Source: J Int AIDS Soc. 2019 Feb 6;22(2):e25242. doi: 10.1002/jia2.25242 (PMC6364490; doi:10.1002/jia2.25242)
Supplement: Supplementary file 1 — Appendix S1: Pre‐exposure prophylaxis(PrEP) knowledge and attitude survey among MSM. [file JIA2-22-e25242-s001.docx]

**Pre-exposure prophylaxis(PrEP) knowledge and attitude survey among MSM**

Before taking the survey, participants have to read and consent with the electronic consent form.

Dear friends:

Thank for your participation in the Pre-exposure prophylaxis (PrEP) knowledge and attitude survey for men who have sex with men (MSM). This survey is jointly supported by the Global AIDS Program of US Center for Disease Control and Prevention (US CDC) China Office, the World Health Organization (WHO) Collaboration Center for HIV treatment at Beijing Ditan Hospital, the Beijing Home of Red Ribbon, and Blued.

Pre-exposure prophylaxis is the use of oral antiviral drugs (ARV) to prevent HIV negative people from contracting HIV infection. WHO guidelines have recommended oral PrEP to people at high risk of HIV since 2012. The WHO Guidelines for the Comprehensive Use of ARV for HIV in 2016 recommend that all people at high risk of HIV infection should use a regimen containing tenofovir (TDF) for PrEP.

Data has showed that the effectiveness of oral regimens containing TDF are similar among people with different genders and ages. The preventive efficacy is strongly correlated with drug compliance. Daily use of oral PrEP is better in terms of have sufficient drug concentration in blood, compliance of treatment and community scale up. Therefore, daily use is generally recommended to people who need PrEP. The purpose of this survey is to understand Chinese MSM’s perception on oral PrEP. The results of the study can be used for the further development of HIV prevention policies in China.

This survey is anonymous, so your personal information will not be at risk of leaking because of your participation. The information you provide will be collected through Wenjuanxing, an online survey platform. Only the authorized survey staff from Beijing Ditan Hospital and Blued can access to the contents of the questionnaire. However, the summary of the survey results will be shared with the technical authorities on HIV prevention and treatment in China, providing a basis for the future policy or research.

You do not have to spend any money to participate in this survey, and we will not offer you any compensation either. Each participator will get the opportunity to take part in the Blued lottery. The information you provide is very important to us and your community. Please fill in the details, thank you for your participation.

| **I have read and understood the above instructions and agreed to participate in**  **this survey.** |
| --- |
|  |
| **1.** Date of the survey: [choosing from drop-down menu of date] |
| _________________________________ |
|  |
| **General information** |
|  |
| **2.** What is your age group? [single choice question] |
| \| ○ under-18s \| ○ 18s~25s \| ○ 26s~30s \| ○ 31s~40s \| ○ 41s~50s \| ○ 51s~60s \| ○ over-60s \|  \| \| --- \| --- \| --- \| --- \| --- \| --- \| --- \| --- \| |
| **3.**Which province are you staying recently for more than 6 months? [choosing from drop-down menu of Chinese provinces list] |
|  |
| **4.** Where is your permanent residence in China? [choosing from drop-down menu of Chinese provinces list] |
|  |
| **5.**You were born as: [single choice question] |
| \| ○ male \| ○ female \|  \| \| --- \| --- \| --- \| |
| **6.**You current identify as : [single choice question] |
| \| ○ male \| ○ female \| ○ not sure \|  \| \| --- \| --- \| --- \| --- \| |
| **7.** What is the highest level of education you have completed? [single choice question] |
| ○ lower than Junior high school     ○ Junior high school     ○ High school     ○ College and above |
| **8.** What is your annual Income (RMB) [single choice question] |
| ○Annual income less than 10,000RMB    ○ Annual incomes is 10,000-30,000RMB    ○ Annual incomes is 30,000-150,000RMB    ○ Annual income more than 150,000RMB |
| **9.**Your Sexual orientation is? |
| _________________________________ |
| **10.** Your marital status is : [single choice question] |
| ○ Single     ○ Cohabitation with a woman     ○ Cohabitation with a man     ○ Married     ○ Married with a woman/lesbian     ○ Separated, Divorced or Widowed  **11.** Your ethnicity? [single choice question]  ○ Han  ○ Non-Han  **12.** How many times did you have sexual intercourse with another man in the past 12 months? If no, please write zero [fill in the blank]    _________________________________ |
| **13.** How many sexual partners do you have in the past 12 months who had anal sex with you? If no, please write zero [fill in the blank] |
| _________________________________ |
|  |
| **14.** Did you ever engage in unprotected anal sex in the past 12 months? [] [single choice question] |
| ○ Yes    ○ No  (Skip to question **19**) |
|  |
| **15.** If Yes，how many sex partners do you have with whom you had unprotected anal sex? |
|  |
| _________________________________ |
|  |
| **16.** Among the sexual partners you had with unprotected anal sex, how many were casual sex partners? [single choice question] |
| ○ All     ○ some    ○no one is casual |
| **17.** How often did you and your unprotected anal sex partner (s) talked about HIV before a sexual intercourse? [single choice question] |
| ○ every time    ○ sometimes     ○ never mention |
| **18.** Do you know the HIV status of your unprotected anal sex partners? [single choice question] |
| ○yes，very clear     ○ yes，partially clear     ○ completely don’t know   ○ hard to answer |
| **19.** When did you test for HIV last time？ [] [single choice question] |
| ○in the past 6 months     ○in the past 6-12 months     ○ in the past 1-5 years    ○ 5 years ago   ○ I have never been tested for HIV (Skip to question **21**) |
| **20.**What was your last HIV testing result? [single choice question] |
| ○ negative    ○ positive    ○ don’t know |
| **21.** Have you ever been diagnosed with any STD in the past 12 months？ [single choice question] |
| ○ Yes    ○ No |
| **22.** When was your last STD examination？[single choice question] |
| ○ in the past 6 months    ○ in the past 6-12 months     ○ 1year ago    ○ I have not been checked for STD |
| **23.** How often did you go to gay entertainment places (such as bars, baths, parks, etc.) in the past 30 days? [single choice question] |
| ○ 4-5 times per week    ○ 1-2 times per week    ○ 2-3 times per month    ○ Once per month or less    ○ never |
| **The following questions are about the cognition and acceptability of PrEP.** |
| **24.** Have you ever used PrEP before? [single choice question] |
| ○ yes    ○ no |
| **25.** Before this survey, have you ever heard that PrEP can prevent HIV infection? [single choice question] |
| ○ Yes, Heard of PrEP  ○ NO (skip to question **27**)  ○ I don’t know (skip to question **27**) |
| **26.** Where did you get information about PrEP? [single question] |
| ○ Medical facilities    ○ Internet    ○ Gay community or CBO    ○ Other gay /friends |
| **27.** Whom do you think should use PrEP to prevent HIV infection? [multiple choice questions] |
| □ HIV/AIDS patient’s partner whose HIV status is negative   □ Men who engage in commercial sexual behavior    □ sex workers    □ People who are afraid of AIDS   □ people who don’t like condom    □ People cannot use condom consistently    □ The role of "0" without anal intercourse    □ people who frequently change sexual partners |
| **28.** If PrEP is available in China, will you use them? [single choice question] |
| ○ No, I will not use (Skip to question 30)   ○ maybe not   (Skip to question 30)   ○ not sure (Skip to question 30)   ○ Maybe yes   ○ Definitely Yes |
|  |
| **29.** Where do you prefer to get the PrEP prescription? [single choice question] |
| ○ Medical facilities     ○ Pharmacies/ internet pharmacies    ○ Gay communities     ○ Vending     ○ Others _________________ |
|  |
| **30.** What are the concerns making you hesitate to use PrEP ?[multiple choice questions] |
| □ Have no risk of getting HIV     □ Doubts on PrEP’s efficacy     □ Worry about PrEP’s side effects     □ Financial burden     □ Have no access to PrEP     □ Inconvenience in taking PrEP everyday     □ Prefer using Condom as a protection for HIV     □ others _________________ |
|  |
