# Supplementary material for: PrEP uptake preferences among men who have sex with men in China: results from a National Internet Survey
Source: J Int AIDS Soc. 2019 Feb 6;22(2):e25242. doi: 10.1002/jia2.25242 (PMC6364490; doi:10.1002/jia2.25242)
Supplement: Supplementary file 2 — Appendix S2: Numbers of participants by provinces/regions. [file JIA2-22-e25242-s002.docx]

Numbers of participants by provinces/regions

Anhui Province: 135

Macau: 3

Beijing: 300

Fujian Province: 122

Gansu Province: 83

Guangdong Province: 456

Guangxi Zhuang Autonomous Region: 139

Guizhou Province: 70

Hainan Province: 28

Hebei Province: 209

Henan Province: 286

Heilongjiang Province:143

Hubei Province: 192

Hunan Province: 193

Jilin Province: 107

Jiangsu Province: 248

Jiangxi Province: 100

Liaoning Province: 169

Inner Mongolia Autonomous Region: 82

Ningxia Hui Autonomous Region: 209

Qinghai Province: 219

Shandong Province: 314

Shanxi Province: 118

Shaanxi Province: 159

Shanghai: 127

Sichuan Province: 238

Taiwan: 1

Tianjin:72

Tibet Autonomous Region: 7

Xinjiang Uygur Autonomous Region :75

Yunnan Province:86

Zhejiang Province:181

Chongqing:133
